# Supplementary material for: Genotype-phenotype correlations of germline mutations in exon 10 of the RET proto-oncogene from 14 MEN2A families of Ethnic Han Chinese
Source: PLoS One. 2025 Sep 11;20(9):e0332136. doi: 10.1371/journal.pone.0332136 (PMC12425180; doi:10.1371/journal.pone.0332136)
Supplement: Attachment — Submitted filename: V1-Response to Reviewers.docx [file pone.0332136.s002.docx]

**Cover Letter** **for revised MS [ID:** PONE-D-24-51352**]**

**To:** Editor, PLoS One

**Reply to Referees' Comments on MS ID PONE-D-24-51352**

**Dear Editor and Reviewers,**

We are very grateful for your constructive and thoughtful comments on our manuscript “Genotype-phenotype correlations of germline mutations in exon 10 of *RET* proto-oncogene from 14 MEN2A families in Ethnic Han Chinese” (PONE-D-24-51352). Those comments are all valuable and very helpful for revising and improving our paper. We have studied comments carefully and have made correction which we hope meet with approval.

**Response to ediror,**

**Q1.** Please ensure that your manuscript meets PLoS One's style requirements, including those for file naming.

**A1.** We have corrected the manuscript’ style according to PLoS One style templates.

**Q2.** Please state what role the funders took in the study. If the funders had no role, please state: "The funders had no role in study design, data collection and analysis, decision to publish, or preparation of the manuscript." If this statement is not correct you must amend it as needed. Please include this amended Role of Funder statement in your cover letter; we will change the online submission form on your behalf.

**A2.** We confirm that the funders had no role in study design, data collection and analysis, decision to publish, or preparation of the manuscript.

**Q3.** PLOS requires an ORCID iD for the corresponding author in Editorial Manager on papers submitted after December 6th, 2016. Please ensure that you have an ORCID iD and that it is validated in Editorial Manager. To do this, go to‘Update my Information’(in the upper left-hand corner of the main menu), and click on the Fetch/Validate link next to the ORCID field. This will take you to the ORCID site and allow you to create a new iD or authenticate a pre-existing iD in Editorial Manager.

**A3.** We confirm that the corresponding author already has an ORCID iD, and it has been successfully validated in Editorial Manager.

**Q4.** Your ethics statement should only appear in the Methods section of your manuscript. If your ethics statement is written in any section besides the Methods, please delete it from any other section.

**A4.** We have deleted any duplicate or misplaced ethics - related content from other sections to ensure compliance with your requirements.

**Q5.** Please include a separate caption for each figure in your manuscript.

**A5.** Thank you for your feedback! We have now added a separate caption for each figure in the manuscript, ensuring that every figure has a clear and accurate description.

**Response to Reviewer 1,**

**Q1**. Enhance the discussion by including more comparisons with non-Asian populations and exploring the molecular mechanisms underlying phenotypic differences.

**A1.** We have incorporated more comparisons and expounded on the possible reasons in our discussion. Compared the tumor-specific penetrance rate of 340 MEN2A patients with exon 10 of *RET* mutations reported in reference 10, the penetrance of MTC in the Asian population is significantly higher than that in the non-Asian population, while the penetrance of PHEO is lower than that in the non-Asian population. It can be inferred that the different penetrance rate of MTC and PHEO between the two populations is caused by the later diagnosis age of MTC and the earlier diagnosis age of PHEO in this study[10]. And the penetrance of MEN2A- MTC/PHEO may be also influenced by differences in race and regional distribution, as well as sample size. It may be also suggested that the same type of *RET* mutation can lead to different clinical phenotypes and disease progression [1,5,13]. The specific molecular mechanism may require further research to elucidate.”

**Q2**. Incorporate more explanatory graphs to highlight differences between genetic subgroups, making the results clearer for readers.

**A2.** We have created additional explanatory graphs to illustrate the differences between genetic subgroups, such as Fig 1and Fig 2. By incorporating these graphs, we hope to enhance the clarity of our results and make it easier for readers to interpret the differences.

**Q3**. Detail potential biases of the retrospective study, addressing limitations in the generalization of findings to broader populations.

**A3.** We have added the sentence“Since this is a retrospective study and the sample size is relatively small, there is a certain degree of bias, such as selection bias and information bias, in the study. It is hoped that multi-center studies can be carried out in the future to increase the generalizability of the results.”in the discussion section to address limitations in the generalization of findings to broader populations.

**Q4**. Expand clinical applicability by providing recommendations for medical practice based on RET genotype, facilitating personalized management strategies.

**A4.** We discussed the potential benefits of personalized management strategies based on RET genotype in the Discussion section from line from 299 to 306: “In addition, our study findings also showed that although there is no significant difference in the age at diagnosis and the penetrance of MTC between patients with C618/C620 mutations and those with C609/C611 mutations, patients with C618/C620 mutations have a higher risk of cervical lymph node metastasis which suggests that in clinical practice, more comprehensive examinations are required for such patients.”

By doing so, we hope to expand the clinical applicability of our study and contribute to the development of more personalized and effective medical practices.

**Response to Reviewer 2,**

**Q1.** On a grammatical level, I noticed several sentences throughout the text with phrasing that felt awkward, if not outright incorrect. Additionally, the authors should consider breaking up long, multi-line sentences into smaller pieces to improve clarity. Overall, I believe the text would benefit from a good line-editing service.

**A1.** We fully acknowledge the issues you pointed out. Regarding the awkward phrasings and potentially incorrect sentences, we have conducted a comprehensive grammar check. We also sought the help from native English speakers to identify and rectify these problems, ensuring the language used is both accurate and natural.

**Q2.** When listing corresponding authors there is no need to reiterate their affiliations, only state preferred methods of communication: mail, phone, etc.

**A2.** The corresponding authors’affiliations have been deleted.

**Q3.** The phrasing “RET mutations within exon 10” throughout the text sounds like “RET” is the name of these specific mutations and not of the gene where they occur. Re-writing it as “exon 10 mutations of RET gene” makes the message more clear.

**A3.** All the“*RET* mutations within exon 10”has been replaced to“exon 10 mutations of RET gene”.

**Q4.** Lack of line and page numbering makes it more cumbersome to make comments on particular parts of the text.

**A4.** The line and page number have been added to the manuscript.

**Q5.** Reference numbers throughout the text shouldn’t be in blue color and uppercase.

**A5.** All the reference numbers have been cancelled blue color and uppercase.

**Q6.** On the title page the authors say that the text contains a single figure and a single table, but attach two figures to the text.

**A6.** This illustration is not necessary, we have deleted it.

**Q7.** Figures should contain legends, did not find them anywhere.

**A7.** We have added figure legends in the last page.

**-Abstract:**

**Q8.** In the sentence “Comparison of the age at diagnosis...” change “there were” to “revealed” for correct grammatical structure.

**A8.** We have changed“there were”to“revealed”.

**Q9.** The sentence “Patients were divided into...” is not grammatically correct, please rewrite for clarity. Also, it is not clear from the sentence if N1 and N0 patients were compared between groups or inside each group of mutation carriers. And font size of “were divided into” for some reason is different from the rest.

**A9.** We have rewrote the sentence to“There was a significant difference in the positive rate of N1 between patients with p.C618/C620 mutations and those with p.C609/C611 mutations. Additionally, there was also a significant difference in the initial serum calcitonin levels between N1 and N0 patients (*P* < 0.05).”

**- Introduction:**

**Q10.** The sentence “Recently, it has been proved...” is not entirely clear to me. Do the authors mean that improve in diagnostics lead to a higher number of identified cases? Or that overall amount of people carrying these mutations keeps increasing for some reason?

**A10.** We have changed it to“Recently, it has been reported that the proportion of patients with exon 10 mutations has increased from 8% to 25% with more and more cases disclosed.”

**Q11.** In the sentence “most studies in the literatures describe only through single family”: 1) I think in the case here using “literature” is more correct and common; 2) “a” should be used instead of through”

**A11.** We have changed it to“However, most studies in the literature describe only a single family or case with exon 10 mutations”

**- Materials and Methods:**

**Q12.** “exon10” - missing space.

**A12.** We have added the space.

**- Results:**

**Q13.** The sentence: “In all, 74 of 133 participants who were found...” does not sound grammatically correct.

**A13.** We have corrected it to“Out of 133 participants, 74 cases carrying the RET germline mutations were diagnosed with MEN2A, including 26 males and 48 females.”

**Q14.** Consider breaking long multi-line sentences into shorter segments for better clarity of the text.

**A14.** We have rewrote the paragraph and broke long multi-line sentences into shorter segments to make the presentation clearer.

**Q15.** “pathologicaal” - extra “a” typo.

**A15.** We have deleted the extra“a”extra.

**- References:**

**Q16.** Reference numbers should have same font color.

**A16.** The font color of the references has been unified.

**Q17.** Adding “[J]” after journal title is not a part of a reference style used by PLOS

**A17.** All the“[J]”after journal title have been deleted.

**Q18.** The volume number should be separated from the year of publication by a semicolon, not a comma.

**A18.** The volume number and the year of publication has been separated from by a semicolon.

**Q19.** The references should include six author names before using et al., not three.

**A19.** We have added another three author names before using et al.

We appreciate for your warm work earnestly, and hope that the correction will meet with approval.

Once again, thank you very much for your comments and suggestions.

Yours sincerely,

Xiaoping Qi
