# Supplementary material for: Genotype-phenotype correlations of germline mutations in exon 10 of the RET proto-oncogene from 14 MEN2A families of Ethnic Han Chinese
Source: PLoS One. 2025 Sep 11;20(9):e0332136. doi: 10.1371/journal.pone.0332136 (PMC12425180; doi:10.1371/journal.pone.0332136)
Supplement: Attachment — Submitted filename: Response to Reviewers.docx [file pone.0332136.s003.docx]

**Reply to Referees' Comments on MS ID PONE-D-24-51352**

**Dear Editor and Reviewers,**

We are very grateful for your constructive and thoughtful comments on our manuscript “Genotype-phenotype correlations of germline mutations in exon 10 of *RET* proto-oncogene from 14 MEN2A families in Ethnic Han Chinese” (PONE-D-24-51352). Those comments are all valuable and very helpful for revising and improving our paper. We have studied comments carefully and have made correction which we hope meet with approval.

**Response to Reviewer 2,**

While the authors have done commendable work in addressing some of the specific points I raised earlier, the main issue of proper article presentation remains unresolved. I have identified multiple typos, grammatically incorrect and run-on sentences, and instances of awkward phrasing that limit the reader's ability to fully understand the article. I would emphasize again the point I have raised in the previous review: the authors should perform a comprehensive line-editing of the manuscript, as it still reads more like an advanced draft than a publication-ready paper.

The issues mentioned above include, but are not limited to, the following:

1) Typos — found in lines: 47, 75, 110, 128, 136, 227, 241, 276, and others.

2) Grammatical errors — in lines: 53, 90–92, 111, 178, 195–196, 210–212, among others.

3) Run-on and awkward sentences — in lines: 242–247, 265–270, 276–280, and more.

4) Figure legends — when embedded in the main text, they should be clearly separated to avoid confusion between the legend and the text itself.

5) There shouldn't be multiple empty lines between "References" title and previous section.

Answer to the

A. We have engaged a professional medical English editor to polish the language of the article and corrected all the linguistic errors you mentioned, and the proof is as follows:

Additionally, I would like to point out a few technical issues in the article:

1) The "Methods: Detection of RET mutation" subsection would benefit from a brief description of the actual methods used, rather than referring readers only to previous articles. The methodology section should stand on its own, at least at a high level.

A1. Thanks for your suggestion. We have we have outlined the methodology. “Peripheral blood samples (5 mL) were collected from all 133 family members, including the probands, and then the Illumina HiSeq 2000 Analyzer was used for targeted sequencing. The targeted sequencing results were further validated by Sanger sequencing using an ABI 3700 Genetic Analyzer (Perkin-Elmer, Fremont, CA)”

2) The authors should elaborate more about ref. 10, since they use its data extensively within the article. At a minimum, the Methods section should briefly explain what ref. 10 includes, why it was chosen for comparative analysis, and which factors could complicate direct comparisons between its data and the findings of this study.

A2. Thanks for your suggestion. We have elaborated more about ref. 10 in the Methods section. “Data from the International RET Exon 10 Consortium, which included 340 patients from 103 families in 15 countries, were used for comparison with this study”

3) The subsection "Results: Outcome of treatment" does not seem to include much information regarding connections between observed genotypes and treatment outcomes. While this section offers useful clinical information, the authors should stay focused on the core topic of the article — the association between RET mutations and oncogenic phenotypes.

A3. Thanks for your suggestion. We have added more information between genotypes and treatment outcomes. “Post-operation, 48 of the 49 patients with MTC were confirmed histopathologically, while the remaining 1 patient (with the C618Y mutation) was confirmed as having CCH. All patients with the C609R, C618G/S/Y and C618S/R were confirmed to have positive lymph nodes. Whereas, the positive rate of lymph nodes in patients with the C611Y and C618R mutations was 7/14 (50%) and 12/16 (75%), respectively.”

4) The figures would benefit from more informative legends. Technical information, such as p-values of various comparisons, should be shown on the chart itself and does not need to be reiterated in the legend. Additionally, the figures would benefit from being in color rather than black and white, to improve readability.

A4. Thanks for your suggestion. We have moved the technical information from the legends to the charts. And the figures have been re-drawn in color.

5) Figure 2 — The y-axis should display the percent (or fraction) of pedigrees rather than absolute counts, since the compared groups have significantly different sample sizes.

A5. Thanks for your suggestion. The y-axis of Figure 2 has been displayed the percent.

6) SI: Medical Ethics Review Report - It is better to attach the translation as PDF, to ensure compatibility and ease of access across different devices.

A6. Thanks for your suggestion. The Medical Ethics Review Report has been attached to the SI as PDF.

We appreciate for your warm work earnestly, and hope that the correction will meet with approval.

Once again, thank you very much for your comments and suggestions.

Yours sincerely,

Xiaoping Qi,

18, Aug, 2025
